# Typhoid Fever Epidemic in Paris

**Published:** 1882-11

**Authors:** 


### TYPHOID FEVER EPIDEMIC IN PARIS.

About the end of September this disease began to exhibit an unusual frequency in the French Capital and at the latest accounts it was prevailing there epidemically. The weekly mortality increased in consequence from 932 for the week ending September 21st, to 1,174 for that ending October 12th, and the mortality from typhoid fever from 53 to 250. The subject was introduced into the French Academy, by M. Marjolin at the Séance of October 24th, when the majority of those who spoke were inclined to attribute its prevalence to the inefficiency of the law relating to unhealthy dwellings. This law enacted in 1850 it seems, has never been enforced. The practical importance of the subject at this time led to its adoption as the order of the day. It will doutless receive very thorough treatment at the hands of the French pathologists and much light may be expected to be thrown upon it by the facts and opinions which will be elicited.—*Md. Med. Journal.*

